# Microincision Vitrectomy Surgery in Vitreomacular Traction Syndrome of Retinitis Pigmentosa Patients

**DOI:** 10.1155/2014/537081

**Published:** 2014-06-09

**Authors:** Enzo Maria Vingolo, Emanuele Gerace, Stefano Valente, Leopoldo Spadea, Marcella Nebbioso

**Affiliations:** ^1^Department of Ophthalmology, Polo Pontino, A. Fiorini Hospital, Via Firenze, 04019 Terracina, Italy; ^2^Sapienza University of Rome, Piazzale Aldo Moro 5, 00185 Rome, Italy; ^3^Department of Sense Organs, Centre of Ocular Electrophysiology, Sapienza University of Rome, Policlinico Umberto I, Viale del Policlinico 155, 00161 Rome, Italy

## Abstract

*Purpose*. To investigate long-term retinal changes after microincision pars plana vitrectomy surgery (MIVS) and internal limiting membrane (ILM) peeling outcome in retinitis pigmentosa (RP) patients affected by vitreomacular traction syndrome (VMT) with higher vitreous surface adhesion or coexisting epiretinal membrane (ERM). *Methods*. Eight RP patients suffering from VMT were evaluated by means of best corrected visual acuity (BCVA), anterior and posterior binocular examination, spectral-domain optical coherence tomography (SD-OCT), MP-1 microperimetry (MP-1), and full-field electroretinogram (ERG), before MIVS and ILM peeling and during the 36-month follow-up. Patients were hospitalized for two days after the surgery. Surgical procedure was performed following this schedule: surgical removal of crystalline lens (MICS), MIVS with 23-gauge sutureless system trocars, core vitreous body removal, and balanced-sterile-salin-solution- (BSS-) air-gas (SF_6_) exchange. *Results*. All patients presented visual acuity (VA) increase after MIVS. None of the patients developed ocular hypertension or vitreomacular adhesions during the 3-year follow-up. MP-1 bivariate contour ellipse area (BCEA) was reduced in its dimensions and improved in all patients demonstrating a better fixation. *Conclusions*. MIVS could be the gold standard therapy in RP patients with VMT and higher vitreous surface adhesion or coexisting ERM if medical therapy is not applicable or not effective.

## 1. Introduction

Retinitis pigmentosa (RP) is a broad group of inherited retinal diseases. It is characterized by photoreceptor degeneration and retinal pigment epithelium (RPE) disorders [1]. RP usually starts from the rods and peripheral cones; successful macula soon gets involved in most of the cases [2]. Night blindness and a progressive constriction of the visual field are the common symptoms. Main features of the overt diseases and classical fundus exam signs of the disorder are waxy pallor of the optic nerve head, thinning of retinal vessels, diffuse granularity with pigment clumping, and bone spicules [3]. Its prevalence is 1/4.000 and it frequently leads to legal blindness [4].

Macula alterations usually involve the inner surface of the retina and they are brought on by inflammatory cells migration in the vitreous body. Frequently several alterations are due to chronic blood retinal barrier breakdown that cause cystoid macular edema (CME) by means of liquid leakage from retinal vessels and interruption of the photoreceptors outer segment [5–8]. These retinal anomalies can be diagnosed with spectral-domain optical coherence tomography (SD-OCT).

Mini invasive vitrectomy surgery (MIVS) is the surgical procedure we used for treating a group of selected patients with vitreomacular traction (VMT) and RP. Nevertheless there are some reasons to avoid this surgical solution in eyes with inherited retinal degenerative diseases [9, 10]. Since RP is a rare disease and not all patients develop harmful complications, MIVS has been reported to be executed only a few times [11]. In fact, in RP patients, intraocular direct light and mechanical direct effect on the retina might further damage inner and outer retina layers and cause disease worsening.

The purpose of this study was to evaluate the morphological and functional results of MIVS associated with ILM peeling in patients affected by RP showing VMT with higher vitreous surface adhesion or coexisting epiretinal membrane (ERM).

## 2. Methods 

In accordance with the Helsinki Declaration, the patients and the controls were informed about all relevant aspects of the surgery and of the use of their data. They signed an informed consent before enrolment. The study protocol was approved by the Ethical Committee of the Sapienza University of Rome.

**Figure 1:**
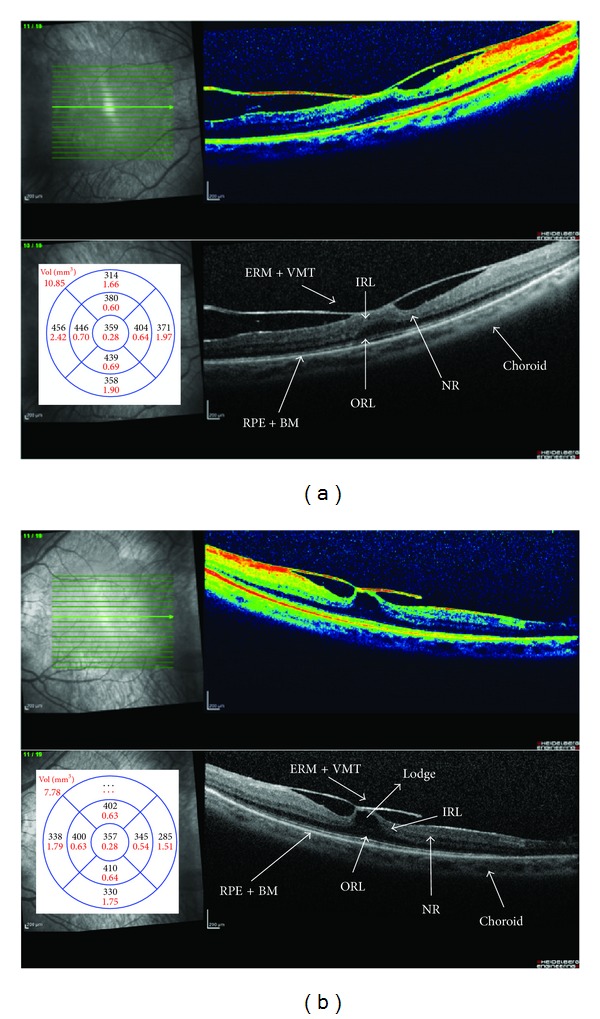
Spectral-domain optical coherence tomography (SD-OCT) of right (a) and left (b) eyes. Retinitis pigmentosa (RP) patients affected by vitreomacular traction syndrome (VMT) with higher vitreous surface adhesion and/or coexisting epiretinal membrane (ERM). Intraretinal macular detachment with hyporeflective and convex lodge observed in the neuroretina (NR) near the surface between inner (IRL) and outer retina (ORL) layers. Bruch's membrane (BM). Retinal pigment epithelial (RPE). Macular average thickness (*μ*m) and volume (mm^3^).

**Figure 2:**
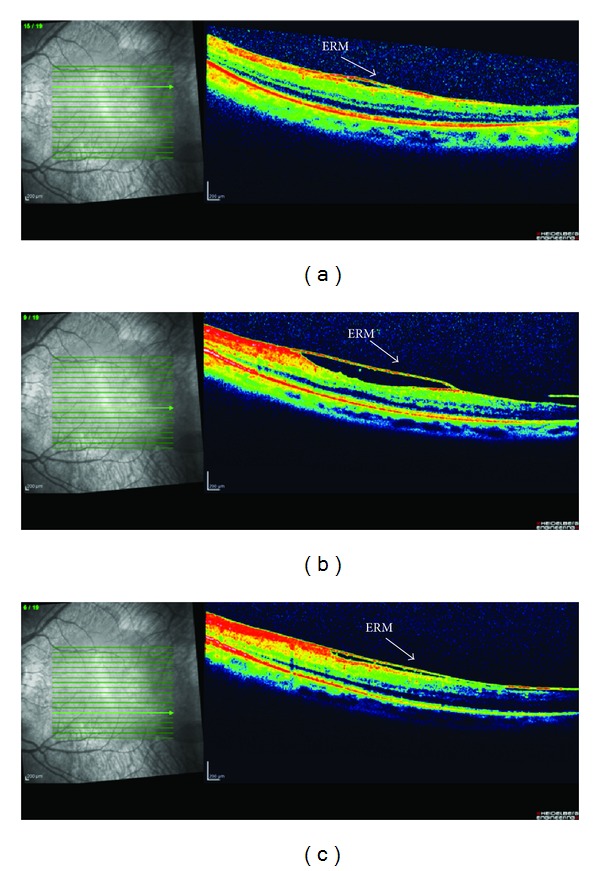
Spectral-domain optical coherence tomography (SD-OCT) of left eye. Retinitis pigmentosa (RP) patient affected from vitreomacular traction syndrome (VMT) and coexisting epiretinal membrane (ERM) in area perifoveal. Scans above (a) and under the fovea (b) and (c).

We enrolled 8 patients, 5 males and 3 females, affected by RP with VMT in eyes with higher vitreous surface adhesion or coexisting ERM (Figures 1 and 2). The mean ± standard deviation age was 51.44 ± 19.82 years (range from 24 to 80 years). They were selected from medical records of more than 2,500 patients of our center for inherited retinal diseases. All patients were followed up with common clinical evaluation for a long time from 3 to 18 years before the surgery. Regarding genetic transmission one patient presented type I Usher's syndrome and 7 rod cone dystrophies of which one was autosomal dominant retinitis pigmentosa (ADRP) with codon 135 rhodopsin gene mutation and 6 patients were affected by sporadic or autosomal recessive retinitis pigmentosa (ARRP). The included patients were unresponsive to conventional medical treatment based on carbonic anhydride inhibitors and nonsteroidal anti-inflammatory drugs (NSAID) or steroids [12]. We suggested to them the surgical procedure in case of visual acuity (VA) worsening and in case of an increase of retinal thickness. Consequently, they underwent 23-gauge MIVS (Accurus vitrectomy system, Alcon Inc., Irvine, CA).

Patients were analyzed to assess their clinical RP status before the surgery. We evaluated

- best corrected visual acuity (BCVA) measured using Snellen and Bailey-Lowson (ETDRS) charts;
- anterior and posterior binocular examination;
- Cirrus HD SD-OCT (Zeiss, Germany) or Spectralis High-Resolution HRA+OCT (Heidelberg Engineering, Germany);
- full-field electroretinogram (Stimulator MV Monitor Mon-Pack 120 Metrovision, Pérenchies, France);
- MP-1 Microperimetry (MP-1) (Nidek Technologies, Padua, Italy), executed in eyes with Snellen BCVA higher than 20/200 (1.0 logMAR) and/or with a good central vision; mean bivariate contour ellipse area (BCEA) has been normalized with a logarithmic transformation and the first standard deviation.

Surgical procedure was performed in 8 eyes with a three-port pars plana vitrectomy under retrobulbar anaesthesia and simultaneous cataract surgery by the same surgeon (EMV).

MIVS has been executed following this procedure.

*Step 1*. Microincision cataract surgery (MICS) in phakic patients for surgical removal of lens: phacoemulsification, aspiration, and IOL implantation.

*Step 2*. Sclerotomy and MIVS with 23-gauge no stitches system trocars.

*Step 3*. Core vitreous body removal.

*Step 4*. Posterior hyaloids, ILM, and ERM peeling with disposable Eckardt forceps.

*Step 5*. Balanced-sterile-salin-solution- (BSS-) air-gas (SF_6_) exchange.

Patients were hospitalized for two days after the surgery. Betamethasone 0.1% eye drops were instilled for 10 days, followed by fluorometholone 0.1% for several weeks. Controls were scheduled at baseline 1, 4, and 18 weeks. Patients were followed up for 36 months.

Pearson's correlation and double tail paired *t* test were used for statistical analysis.

## 3. Results

All patients after the surgical procedure denoted a subjective improvement of visual function, reporting a sharper image, less cloudiness, and, moreover, a wider perception of visual field.

At baseline, BCVA was ranging from light perception (LP) to 20/100 at Snellen charts (0.52 logMAR). After 18 weeks BCVA was ranging from 20/200 (1.0 logMAR) to 21/30 (0.15 logMAR), with statistical significance value (*P* = 0.003). SD-OCT showed a considerable reduction in central retinal thickness (CRT) that presented at the baseline the value of 381.75 ± 94.27 *μ*m and at the end of the follow-up was of 242.75 ± 28.90 *μ*m, showing at statistical analysis a significant value of *P* = 0.005.

BCEA was reduced in its dimensions and improved in all patients demonstrating a better fixation. BCEA ranged from a mean value of *r* ^2^ = 1.165 at baseline to *r* ^2^ = 0.6 after 18 months.

During the follow-up period, no complication due to the surgical procedure was observed in all eyes. Minor postsurgical complications were reported and, particularly, 2/8 patients developed conjunctival chemosis and periorbital hemorrhages due to local anesthesia. SF_6_ gas bubble was reabsorbed within 4 weeks. During the hospitalization, 2 days after the surgical procedure, 2/8 patients showed periorbital pain that disappeared after 24 hours using NSAID.

## 4. Discussion 

**Figure 3:**
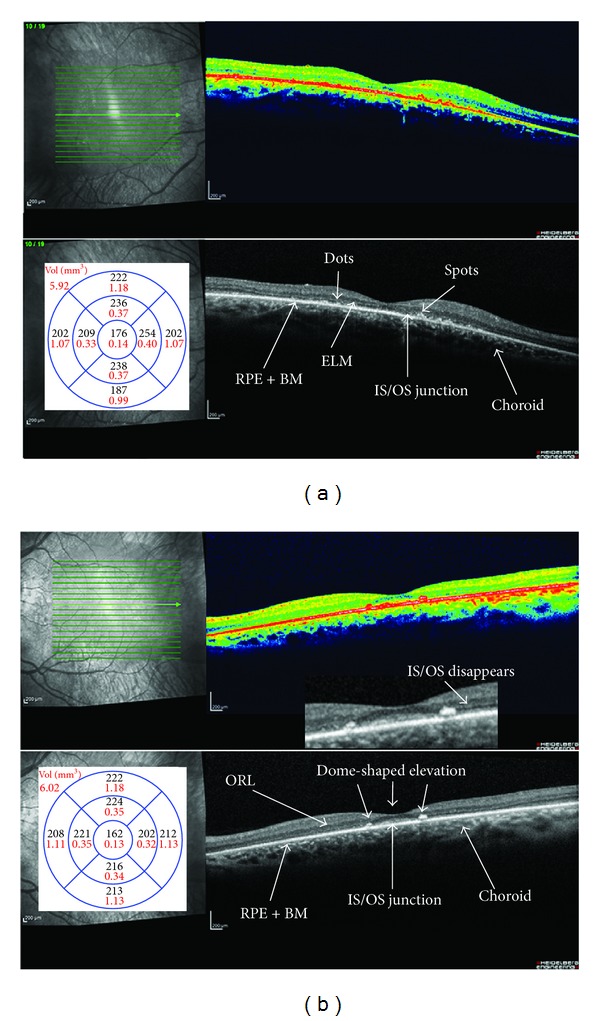
Spectral-domain optical coherence tomography (SD-OCT) of right (a) and left (b) eyes. Retinitis pigmentosa (RP) patients after mini invasive vitrectomy surgery (MIVS) with internal limiting (ILM) and epiretinal membrane (ERM) peeling. Intraretinal macular detachment and the convex lodge are disappearing. The macular thickness is reduced, particularly, the outer retinal layers and retinal pigment epithelial** (**RPE) are thinning and impairing. Right eye (a). There are hyperreflective dots and spots in and around the external limiting membrane (ELM), inner segment/outer segment (IS/OS) junction, and RPE. Left eye (b). The length of the preserved IS/OS junction is between the limits that correspond to the two dotted lines with hyperreflective material in the outer retinal layers (ORL) causing dome-shaped elevations. Magnified view of the border where the IS/OS line disappears. The absence of the photoreceptor integrity line outside this central zone explains the patient's symptoms of very poor night vision and limited side vision. These elevations appear to correspond to pigment clumping seen on fundoscopy. The photoreceptor integrity line is present under the macula but fades outside the macula, a pattern characteristic of rod degeneration.

The purpose of this study was to evaluate the morphological and functional results of 8 RP patients affected from VMT by means of MIVS and MICS associated with ILM peeling. All subjects denoted an increase of VA, a wider perception of visual field, improvements of BCEA, and a considerable reduction in CRT to the SD-OCT after surgery (Figure 3).

Studies in the literature rarely investigated surgical approach in patients affected by RP with VMT. Our study is one of the few reports of this kind of treatment and the results are promising for further studies. Surgery therapy could be useful if maximal medical therapy is not effective or not applicable and there is a worsening of VA associated with an increasing of retinal thickness.

It has already been reported that vitrectomy and ILM removal improved VA in patients with RP reducing macular edema [13]. Furthermore executing MIVS in RP patients without ILM peeling showed a reduction of BCVA [14]. Our patients underwent ILM peeling and their BCVA improved. A late contraction of ILM, indeed, might cause a vision reduction because of epiretinal membrane formation. Besides, its peeling could prevent the development of a macular hole [15]_._ According to some authors it is clear that removing tractive membranes from retinal surfaces may help to reconfigurate the linearity of photoreceptors and all residual inner retinal cells leading to better visual performances improving receptive fields' organization. In fact, eyes with a higher adhesion between vitreous surface and retina or coexisting ERM, a pars plana vitrectomy should be performed with ILM peeling [7].

Furthermore, removal of vitreous body with floating cottonball-like condensations and floccules may help in obtaining better VA and or recovery of pre-VMT visual function [16].

Moreover, we did not execute ILM and dying because ICG is likely to be toxic for the retina [17, 18], Brilliant Blue Peel product was not available in our department for authoritative reasons, absence of ILM was postoperatively confirmed with OCT scans, and from a bare surgical point of view, ILM was really thick and easy to remove in a single large piece covering the whole posterior pole exceeding retinal vessels.

A study showed a significant improvement in anatomical outcome of the macula and VA after surgery observed in three of the patients with RP and MH, indicating that vitreous surgery is proper therapy when a diagnosis of VMT or CME in an RP patient is well established [11]. Some authors stated that preservation of photoreceptors after surgery may be due to the diffusion of cytokines and growth factors originated from the stimulated ciliar body, iris, and retina [19–22]. They are basic fibroblast growth factor (bFGF) [23], neurotrophic growth factor (CNTF) [24], brain-derived neurotrophic factor (BDNF) [25], nerve growth factor (NGF) [26], and lens-epithelium-derived growth factor (LEGF) [27]. This evidence has been proven by a study of Mahmoud et al. They showed that a combined lensectomy and vitrectomy procedure in P347L transgenic pigs was associated with retention of a significantly greater number of outer nuclear layer nuclei than in unoperated fellow eyes [28].

In our experience, after a 3-year follow-up our patients did not develop ocular hypertension in agreement with other authors' findings about long-term IOP changes after combined cataract surgery and vitrectomy. They reported that gas tamponade did not affect postoperative IOP and only a few cases showed an IOP increase [29–33].

On the other hand, the development of narrow-gauge transconjunctival vitrectomy systems has improved the visual recovery following surgery. We think that, at this stage in our knowledge, removal of any vitreomacular traction is probably the main reason for visual improvement and better fixation postoperatively.

In summary, MIVS resulted in better visual and anatomic outcomes when treating RP patients in eyes with higher VMT or coexisting ERM. Studies of other cases and longer longitudinal observation for such patients are required to understand the pathogenesis and factors affecting prognosis for these RP patients.
